# Congenital self‐healing reticulohistiocytosis: An atypical presentation acquired in a 10‐month‐old

**DOI:** 10.1002/ccr3.6227

**Published:** 2022-08-18

**Authors:** Christopher S. Yuki, Patrick J. Young, Steven Ohsie, Xuan Nguyen

**Affiliations:** ^1^ Midwestern University Arizona College of Osteopathic Medicine Glendale Arizona USA; ^2^ Affiliated Pathologists Medical Group / Binder Institute of Pathology Torrance California USA; ^3^ Dermatology Solutions Gilbert Arizona USA

**Keywords:** CSHR, dermatology, LCH, pathology

## Abstract

Congenital self‐healing reticulohistiocytosis of Hashimoto and Pritzker (CSHR) is a rare, benign form of Langerhans Cell Histiocytosis (LCH) that presents at birth and involutes by 6 months of age. We present an atypical case of CSHR with the first onset at 7 months of age, treated with surgical excision.

## Graphical abstract

Congenital self‐healing reticulohistiocytosis (CSHR) presents at birth; however, in this case, it is acquired at 10 months of age which is atypical. CSHR is benign and it must be differentiated from malignant form of Langerhans Cell Histiocytosis.

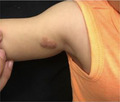

## 1 INTRODUCTION

Langerhans cell histiocytosis (LCH), formerly referred to as Histiocytosis X, is a proliferative disorder of Langerhans cells with a spectrum of clinical presentations with varying prognosis. These myeloid neoplasms of dendritic cells are characterized under electron microscopy by the presence of Birbeck granules resembling that of “tennis rackets.” LCH has been categorized into four main clinicopathologic types: congenital self‐healing reticulohistiocytosis of Hashimoto‐Pritzker disease (CSHR), multifocal multisystem LCH, unifocal LCH, and multifocal uni‐system LCH (Table 1). Multifocal multisystem LCH, previously called Letterer–Siwe disease, is seen in infants and young children less than two years old with clinical findings of seborrheic‐like skin eruptions on the trunk and scalp and with systemic manifestations of hepatosplenomegaly, infiltration of bone marrow with possible pancytopenia, and lytic bone lesions which portends a poor prognosis. Unifocal LCH, also known as eosinophilic granuloma, presents as a localized, more indolent, and benign form of LCH in children greater than 5 years old. Systemic features of eosinophilic granuloma include bony lesions that are commonly seen on radiographic findings of the skull. Multifocal uni‐system LCH, previously known as Hand–Schuller–Christian, is a triad of calvaria bone lesions, diabetes insipidus, and exophthalmos.

**TABLE 1:** This table compares and contrasts the differences in age onset, clinical presentation, systemic manifestations, histology, prognosis, and treatment for the conditions CSHR, Letterer–Siwe disease, Hand–Schiller–Christian disease, and Eosinophilic granuloma

Conditions	Age of Onset	Clinical Presentation	Systemic Manifestations	Histology	Prognosis	Treatment
Congenital self‐healing reticulohistiocytosis	At birth or neonatal period (before 7 months)	Diffuse, localized, or singular lesions; typically reddish or brown in bullous or vesicular shape	Typically resolve in a few months; systemic involvement is rare	Langerhans cells in epidermis and dermis; mixed infiltrate; Birbeck granules on electron microscopy; stains positive for S‐100, CD1a, and CD68	Benign	No specific treatment required aside from topical management for blisters and erosions
Letterer–Siwe Disease	Typically young children <2 years	Multiple papules distributed across the scalp, face, trunk, and buttocks; lesions can be crusted or hemorrhagic	Visceral and bone lesions such as hepatosplenomegaly, lymphadenoapthy, osteolytic lesions; can also affect the pulmonary system, gastrointestinal tract, and hypothalamus	Langerhans cells with mixed infiltrate; Birbeck granules on EM; stains positive for S‐100, CD1a, and CD68; fewer foamy cells than Hand–Schuller–Christian disease	Poor; 5‐year survival rate is 50% with treatment^14^	Vincristine, vinblastine, cyclophosphamide, and steroids; Antimetabolites suggested^15^
Hand–Schuller–Christian disease	Children 2–6 years	Resembles Letterer–Siwe disease or present with papulonodular or granulomatous ulcerations in intertriginous aras; can have an xanthomatous appearance	Diabetes inspidius, bilateral exopthalmos, osteolytic bone lesions (typically the skull), and mucocutaneous lesions	Langerhans cells with mixed infiltrate; Birbeck grandules on EM; stains positive for S‐100, CD1a, and CD68; more foamy and giant cells than Letterer–Siwe disease	Poor, 30% mortality rate^16^	Surgical curettage, excision, or radiation with supplementary intralesional corticosteroids for localized lesions; systemic chemotherapy for multisystemic disease^17^
Eosinophilic granuloma	Variable; primarily in young adults in 40s to 50s, but occasionally in 20‐30 s^18^	Skin lesions are rare; noduloulcerative lesions found in the mouth, perineal, perivulvular, or retroauricular regions	Primary osteolytic bone lesions; while benign may involve calvarium, ribs, pelvis, scapulae, vertebrae, and long bones	Langerhans cells with mixed infiltrate; Birbeck granules on EM, stains positive for S‐100, CD1a, and CD68; few foamy cells, and proliferative infiltrate with eosinophils, histiocytes, and giant cells	Variable	Surgical curettage, sometimes with low‐dose radiation; intralesional glucocorticoids useful adjuncts to therapy^19^

CSHR was first described in 1973 by Hashimoto and Pritzker. This entity is unique from the other variations of LCH due to its benign nature and presentation at birth with involution in the first few months of life.^1^ It usually presents at birth as a solitary, violaceous, papule, or nodule with a central erosion and rolled borders resembling a button.^2^ Histologically, LCH and CSHR are Langerhans cell proliferations that show Birbeck granules on electron microscopy. Birbeck granules are present in 10%–30% of CSHR cases, whereas it present in 50% of other variants of LCH.^3^ Due to the histological similarities between LCH and CSHR, systemic evaluation for extent of disease is necessary to differentiate the benign form of CSHR from the malignant form of LCH.^4^ Herein, we present a unique case of CSHR which occurred past the congenital period with onset at 7 months and diagnosis at 10 months treated with surgical excision to prevent recurrences.

## 2 CASE REPORT

A 10‐month‐old male patient born full term via vaginal birth, otherwise healthy, presented to the dermatology clinic with several 4–8 mm, pink, shiny, grouped, raised, dome‐shaped, umbilicated papules with a central dell located on the right anterior proximal arm present for three months. The clinical differential diagnosis included molluscum contagiosum and multiple epidermal inclusion cysts. Hydrocortisone 2.5% cream was prescribed to be applied twice daily to the affected area for treatment of molluscum dermatitis.

**FIGURE 1:**
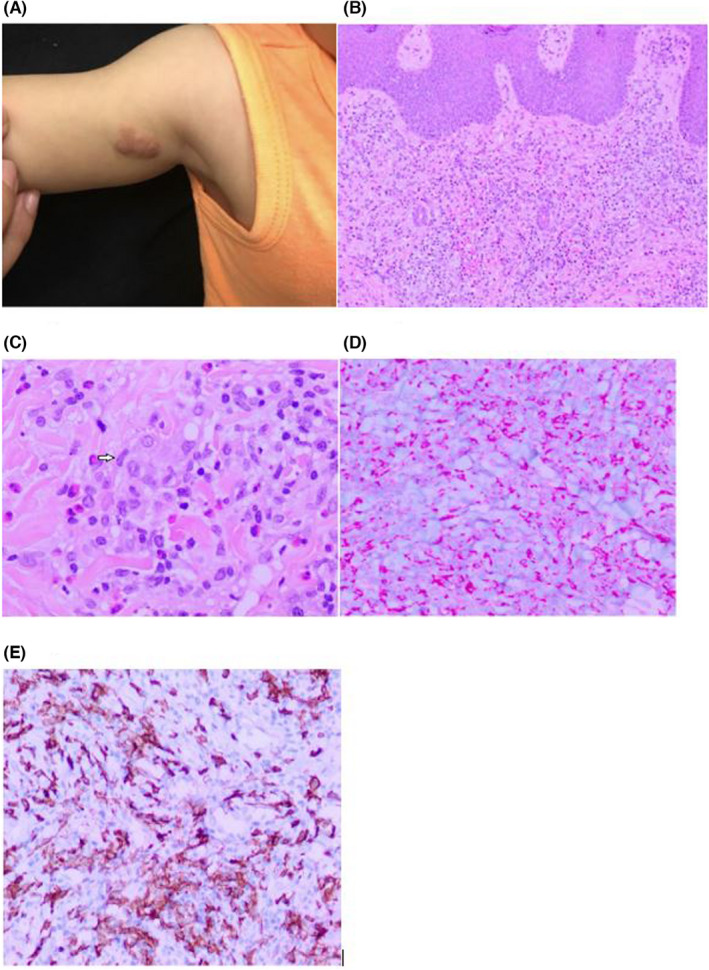
06/19/2019. (A) There is a firm, painless, subcutaneous nodule distributed on the right anterior proximal upper arm. H&E‐stained slides (B,C) show a diffuse dermal infiltrate of histiocytoid cells within a background of inflammatory cells including prominent eosinophils. Focal “kidney‐bean” shaped nuclei can be seen (C). The Langerhans cells show positive staining for S100 (D) and CD1a (E). S100 and CD1a are negative in other histiocytic proliferations

At one‐week follow‐up, the lesion had spread and the scattered, pink papules had coalesced into a larger confluent plaque (Figure 1A). An 8 mm biopsy was performed for the following differential diagnosis: myofibromas, spiradenomas, leiomyomas, and mastocytomas. Pathology showed a histiocytic proliferation with eosinophilia. Immunohistochemistry (IHC) highlighted numerous Langerhans cells that were S‐100+ and CD1a+. At least 30% of inflammatory cells were Ki‐67+. The inflammatory cells also included a mixture of CD3+ T cells, with a predominance of CD4+ T cells over CD8+ T cells, CD20+ B cells, and scattered CD68+ histiocytes. The infiltrate was CD34‐, TDT‐, and SOX‐10‐. Inflammatory cells were positive for stem cell biomarker CD117, as well as mast cells that were tryptase positive. The histological interpretation (Figure 1B–E) were indicative of Langerhans cell histiocytosis (LCH). The following laboratory tests were done: a complete blood count with a differential, liver function tests, urine osmolality, to evaluate for systemic disease. The workup also included a complete skeletal survey to look for any lytic bone lesions along with a chest X‐ray (CXR) to make sure there was no systemic involvement, which is seen in the malignant form of LCH. Additionally, our patient was also referred to pediatric oncology for any additional workup needed to rule out malignant LCH. The systemic workup was all negative and oncology concluded that the lesions were the benign form of LCH. Clinically and histologically, it was concluded that our patient had congenital self‐healing reticulohistiocytosis of Hashimoto and Pritzker (CSHR).

At one‐month follow‐up, our patient was noted to have a pink to brown papule adjacent to the biopsy scar on the right anterior proximal upper arm. Despite the benign nature of CSHR, a surgical excision with clear margins was performed to ensure that there would be no recurrences given the atypical presentation at 7 months of age in our patient. The histology of the surgical excision had the same features as that of the previous biopsy. Hence, we concluded that the recurrent growth was that of CSHR. Our patient was instructed to have clinical follow‐up with dermatology and oncology. Oncology evaluation and subsequent 3‐month follow‐up showed no recurrences, further supporting the diagnosis of CSHR. Three years post‐op, our patient remains well without lesions.

## 3 DISCUSSION

Congenital self‐healing reticulohistiocytosis (CSHR) differs from other forms of Langerhans cell histiocytosis (LCH) because of its benign involuting nature and presentation at birth rather than in early infancy or childhood. CSHR commonly presents with a solitary red‐brown papule or nodule with a button‐like central erosion.^5^ Unique to our patient is the initial onset of CSHR at seven to 10 months of age rather than at birth.

While the various forms of LCH share similar histopathological findings, their pathogenesis has not been clearly elucidated. Eosinophilia, as well as CD1a+ and S‐100+ stains, are common presentations of LCH. Other immunostains for LCH include IFN‐ γ, Ki‐67, PHH3, and E‐cadherin. IFN‐γ, which is not expressed by benign Langerhans cells, is present in all forms of LCH, thus exacerbating recurrence and spread in the benign forms.^6^ Ki‐67, a biomarker for neoplastic growth and proliferation, directly correlates with tumor burden and is found in all types of LCH.^7^ PHH3, a mitosis‐specific antibody, is also an indicator for mitotic proliferation.^8^ Loss of E‐cadherin is often associated with a worse prognosis in LCH.^9^ LCH variants share similar histology and IHC; however, due to the clinical presentations being different in the types of LCH, clinico‐histological correlation is important as the formerly known type of LCH called Letterer–Siwe can portend worser prognosis and is in fact malignant. Given that Langerhans cells play a pivotal role in the immune system, the disease is immunologically modulated. However, because of the similar histology between all variants of LCH, the mechanism of pathogenesis is still unclear.^10^

CSHR is a congenital, self‐healing disorder that does not require medical treatment, but rather close clinical follow‐up after diagnosis so that malignant LCH is excluded. Lesions can be multiple or solitary, though this does not ultimately affect involution. However, in the event of malignant LCH, treatment is necessary. Treatment for LCH includes prednisone and chemotherapeutic drugs such as vinblastine.^11^ Non‐systemic treatments include topical 20% nitrogen mustard and Psoralen ultraviolet light A (PUVA).^12^ Thalidomide has been reported as a potential therapeutic agent due to its inhibition of tumor necrosis factor‐alpha and studies have shown thalidomide also impairs proliferation of LCH.^13^

With complete excision removal of the CSHR, our patient's lesion did not recur at the two‐month follow‐up nor at the six‐month follow‐up with dermatology and oncology; follow‐up with dermatology and oncology are important nonetheless. Full workup to evaluate for systemic involvement is integral for the diagnosis of CSHR especially in our case due to the delayed onset beyond the congenital age, and also because the aggressive variants of LCH mimic CSHR histologically. Both concerns addressed above applied to our patient's case. CSHR rarely presents after birth, and recurrent cases may be characterized by malignancy.^2^ Therefore, recognizing LCH as a spectrum of disease is of utmost importance to differentiate the benign from that of the malignant variants of LCH.

Our patient's unique presentation of CSHR with onset at 7 months of age raises a concern for unreported cases of CSHR that arise after birth. This issue is further exacerbated by the similar clinical and histopathological findings between CSHR and other forms of LCH. Thus, it has been speculated that CSHR may occur more frequently than reported.^3^ However, there are no adverse effects on morbidity or mortality because the disease is self‐healing. Overall, it is still necessary to recognize and monitor CSHR, especially when it manifests after birth.

## AUTHOR CONTRIBUTIONS

Christopher Yuki is the main author and did majority of the writing. Patrick Young helped write the paper. Dr. Ohsie is the pathologist who read the slides. Dr. Nguyen is the overseeing physician.

## CONFLICT OF INTEREST

The authors have no conflict of interest to declare.

## PRIOR PRESENTATION

This case has not been presented in any prior conferences.

## CONSENT

Written informed consent was obtained from the patient to publish this report in accordance with the journal's patient consent policy.

## Data Availability

None
